# Defining the glucosylceramide population of *C. elegans*

**DOI:** 10.3389/fphys.2023.1244158

**Published:** 2023-09-13

**Authors:** Mark A. Xatse, Carissa Perez Olsen

**Affiliations:** Department of Chemistry and Biochemistry, Worcester Polytechnic Institute, Worcester, MA, United States

**Keywords:** glucosylceramide (GlcCer), sphingolipid, *C. elegans*, mass spectrometry, lipidomic analysis

## Abstract

Glucosylceramides (GlcCer) are lipids that impact signaling pathways, serve as critical components of cellular membranes, and act as precursors for hundreds of other complex glycolipid species. Abnormal GlcCer metabolism is linked to many diseases, including cancers, diabetes, Gaucher disease, neurological disorders, and skin disorders. A key hurdle to fully understanding the role of GlcCer in disease is the development of methods to accurately detect and quantify these lipid species in a model organism. This will allow for the dissection of the role of this pool *in vivo* with a focus on all the individual types of GlcCer. In this review, we will discuss the analysis of the GlcCer population specifically in the nematode *Caenorhabditis elegans*, focusing on the mass spectrometry-based methods available for GlcCer quantification. We will also consider the combination of these approaches with genetic interrogation of GlcCer metabolic genes to define the biological role of these unique lipids. Furthermore, we will explore the implications and obstacles for future research.

## Introduction—The biological role of glucosylceramides

Sphingolipids are a class of lipids that play crucial roles as structural components and signaling molecules (Merrill et al., 2009; Merrill, 2011). Sphingolipids are a complex and varied class of lipids characterized by the presence of an amino sphingoid base (Peng et al., 2017). Although they are critical components within the cell, they compromise a fairly small fraction of the total lipid in a cell, especially in comparison to the phospholipid and neutral lipids within an animal (Quinville et al., 2021). Sphingolipids can be further broken into distinct classes including glucosylceramides (GlcCer). Specifically, a GlcCer is a sphingolipid that is produced from the addition of glucose to ceramides via a β-glycosidic bond (Merrill, 2011). GlcCers can be further derivatized to produce other complex glycolipids by specific glycosyltransferases such as BRE enzymes, therefore, GlcCers are considered vital intermediates of sphingolipid metabolism (Merrill, 2011). For example, the BRE enzymes were named based on a *Bacillus* toxin resistance phenotype and have been further characterized to act in glycolipid synthesis ultimately producing complex glycolipids in nematodes and in arthropods (Griffitts et al., 2005; Zhang et al., 2011; Watts and Ristow, 2017).

GlcCers have been found to influence many diverse cellular processes such as cell growth and proliferation, neuronal development, nutrient sensing, cell signaling, apoptosis, intracellular transport, and protein sorting (Aerts et al., 2019; D’Angelo et al., 2013; Iwabuchi et al., 2010; Lalazar et al., 2009; Sillence et al., 2002; Van Meer et al., 2003). However, the complexity of the GlcCer metabolism and the variety in the GlcCer pool has left many unanswered questions about specific GlcCer function and activity *in vivo*. GlcCer are found throughout in animals, plants and fungi, and GlcCer makeup can vary between species (Reza et al., 2021). This variability suggests that the roles and functions might differ as well. The focus of this review is on the specific genes and pathways that metabolize GlcCers in the nematode, *C. elegans.* The nematode, *Caenorhabditis elegans,* has become a useful model to interrogate the bioactivity of lipids because of the ease of genetic dissection of metabolic pathways within the nematode (Kamath and Ahringer, 2003; Zhang et al., 2015). In *C. elegans*, multiple genes can be targeted to impair GlcCer production including *elo-5,* an elongase responsible for the production of monomethyl-branched fatty acids (mmBCFAs) needed to generate the sphingoid base essential for GlcCer production in nematodes (Kniazeva et al., 2004; Kniazeva et al., 2015; Vieira et al., 2022). Emerging research over the years using the model organism *Caenorhabditis elegans*, has provided some insight into the mechanistic role of this unique class of sphingolipids. This review will summarize the key roles of GlcCer uncovered using the *C. elegans,* elucidate challenges in studying GlcCer, and future perspectives of this field.

## Glucosylceramide synthesis and degradation In *C. elegans*

**FIGURE 1:**
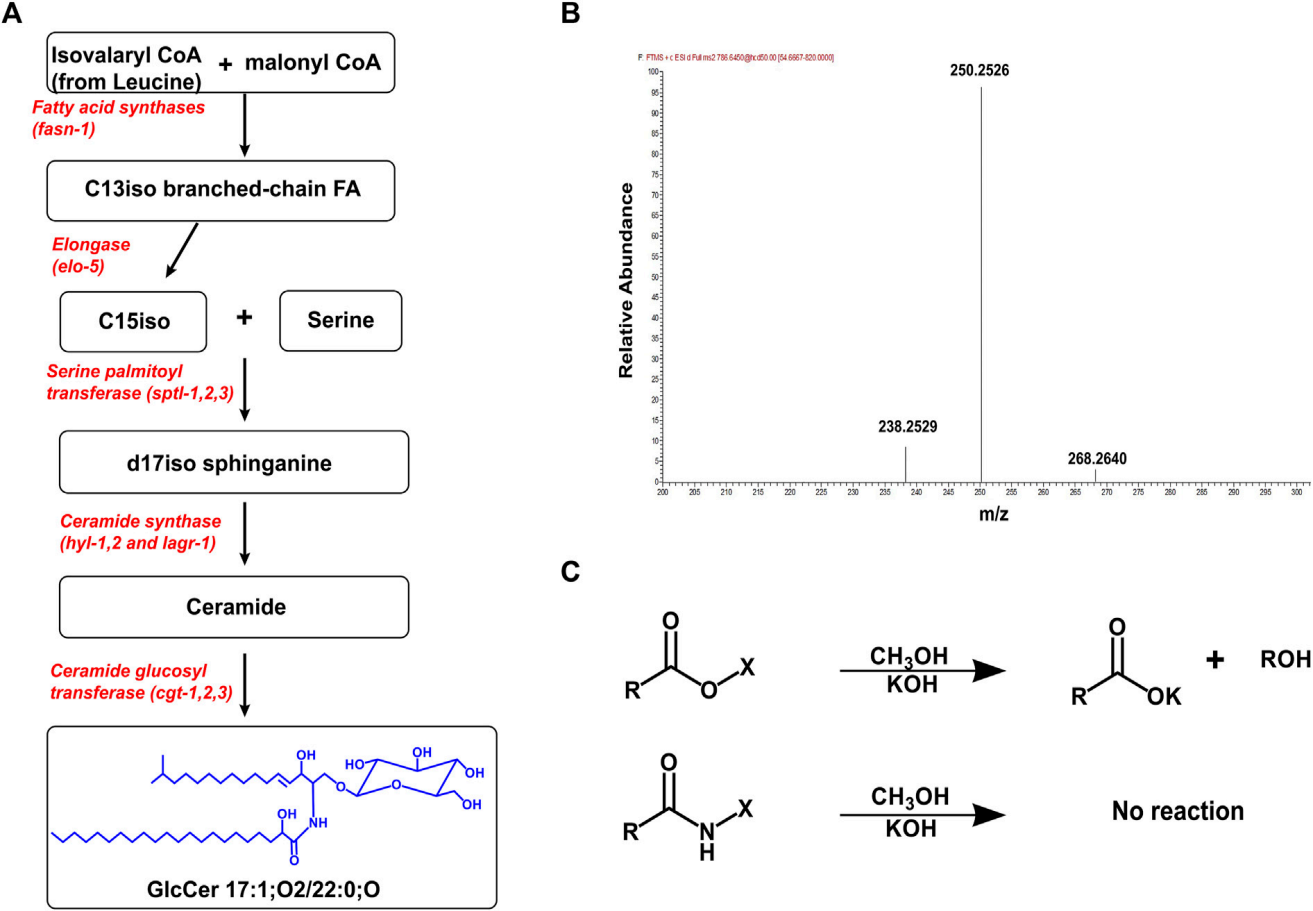
Production, Purification, and MS analysis of GlcCer. **(A)** The pathway for producing GlcCer begins from the production the C15iso fatty acid by fatty acid synthase and elo-5. The enzyme *sptl-1* catalyzes the combination of a C15iso (mmBCFAs) with serine to form the d17iso sphingoid backbone. Different ceramide synthases selectively add fatty acyl chains to the sphingoid backbone to produce various ceramide species. Finally, CGT adds a UDP-glucose to the ceramide molecule to produce a GlcCer **(B)** The mass spectrum showing the MS2 scan for GlcCer 17:1; O2/22:0;O ([M+H] = 786.6465). The fragmentation of GlcCer (and Cer) generates a triplet peak with mass (m/z) 238.2529, 250.2529, and 268. 2640 which is characteristic of a C17:1 sphingoid base found in *C. elegans* (Hänel et al., 2019). **(C)** Sphingolipids including GlcCer are purified from other ester lipids by incubating in 1M KOH (in methanol) for 2 h. This cleaves ester linkages in phospholipids and glycerolipids eliminating chromatographic interference of these lipids during analysis.

In *C. elegans*, the synthesis of GlcCer has been elucidated and begins with the enzyme ELO-5 which produces two monomethyl-branched fatty acids called C15iso (13-methyl myristic acid) and C17iso (15-methyl hexadecanoic acid) (Kniazeva et al., 2004; Watts and Ristow, 2017). C15iso combines with serine to form the ceramide backbone (d17iso sphinganine) via the enzyme serine palmitoyl transferase (SPTL) (Hannich et al., 2017; Watts and Ristow, 2017). In *C. elegans*, there are three ceramide synthases, HYL-1, HYL-2, and LAGR-1 that add a fatty acid to the sphingoid backbone via an amide linkage to form ceramides (Menuz et al., 2009; Mosbech et al., 2013). The fatty acids can range in chain length from 18 to 27 carbons with 0 or 1 double bond, and the specific ceramide synthase used confers chain specificity to sphingolipids (Cheng et al., 2019; Hänel et al., 2019; Xatse et al., 2023). For example, the ceramide synthase, HYL-1, adds fatty acyl chains of 24–26 carbons, whiles HYL-2 adds 20–22 carbon fatty acyl chains to the sphingoid backbone (Mosbech et al., 2013). Finally, the addition of UDP-glucose to an existing ceramide molecule by ceramide glucosyltransferase (CGT) produces glucosylceramide (Figure 1A). In *C. elegans*, there are three ceramide glucosyltransferases, CGT-1, CGT-2, and CGT-3 which are expressed and function in the intestine (Marza et al., 2009; Nomura et al., 2011).

Using mutation or RNAi of each of the genes involved in GlcCer synthesis has probed the biological role of GlcCer in the nematode and has identified an essential role for GlcCer in development as depleting glucosylceramides leads to developmental defects and larval arrest (Lochnit et al., 2005; Laplante and Sabatini, 2009; Nomura et al., 2011; Zhu et al., 2013; Liu and Sabatini, 2020). Specifically, mutations in *cgt-3* lead to embryonic defects and reduced brood size. Nomura et al. (2011) showed the *cgt-3 (tm504)* mutant has abnormal oocytes and eggs that have disrupted membranes and nuclei which accounts for the decreased brood size in this animal. In addition, double mutant *cgt-1; cgt-3* arrest at the L1 stage are uncoordinated, and eventually die. Interestingly deleting *cgt-2* by mutation or RNAi does not exacerbate early larval arrest phenotype in *cgt-1; cgt-3* double mutant suggests that *cgt-2* is not required for the development.

The use of *C. elegans* has allowed for the spatial dissection of GlcCer metabolism. For example, the enzymes, CGT-1 and CGT-3, are highly expressed in a unique subset cell in *C. elegans*, specifically the pharyngeal intestinal valve (PIV), anterior and posterior intestinal cells, intestinal rectal valve (IRV), and the rectal gland cells (RGCs) (Marza et al., 2009). Intriguingly, expressing CGT specifically in this subset of cells can rescue the larval arrest and defects of *cgt-1;cgt-3* suggesting that the activity of CGT is required in only a small subset of cells. Further studies define that improper feeding and deficient cholesterol mobilization contribute to developmental defects and arrest (Boland et al., 2017). Although cholesterol is not synthesized *de novo* in *C. elegans*, it is required for growth and larval development and this study shows that GlcCers are precursors to the synthesis of a novel class of glycosphingolipid known as phosphoethanolamine glucosylceramides (PEGCs), that regulate cholesterol utilization in *C. elegans*. GlcCer deficient animals (*cgt-1; cgt-3, and elo-5*) have reduced levels of PEGC, and supplementing the diet with high levels of cholesterol (2.6 mM) can also rescue the larval arrest cgt*-1; cgt-3 and elo-5* mutants suggesting that the role of GlcCer may be in the mobilization and regulation of cholesterol. There is remarkable homology between sterol regulation in *C. elegans* and in mammals, but there has not been an established role for PEGCs in mammals (Boland et al., 2017). It is possible the PEGCs have not been detected in mammals due to their low abundance or that another molecule acts in a similar manner.

GlcCer levels can be impacted by the rates of production along with the rates of degradation. Although the studies described here largely focus on GlcCer production, it will be important to consider degradation pathways in future studies. GlcCer can be degraded into their parent compound via a salvage pathway. Briefly, GlcCer is broken down to ceramide by glucosyl ceramidases (*gba-1,2,3,4*). The ceramide can then be further degraded to the sphingoid backbone by ceramidase (*asah-1*), and the sphingoid base is phosphorylated by sphingosine kinase (*sphk-1*) to produce sphingosine 1-phosphate, an important signaling molecule. Sphingosine 1-phosphate can be further broken down to ethanolamine phosphate and a C17iso aldehyde by sphingosine 1-phosphate lyase (*spl-1*), which can be used to produce other lipids. The degradation pathway is also critical for nematode health. For example, the loss of SPL-1 causes severe defects in both the gut and the reproductive tract of *C. elegans* (Zhang et al., 2015; Watts and Ristow, 2017; Wang et al., 2021).

The GlcCer population in nematodes is different from mammals despite conservation of GlcCer synthesis and degradation enzymes (Table 1). Sphingoid base of varying length have been found in mammalian systems and are enriched in specific tissue/organs. The common sphingoid base are 18 carbon (d18:0, d18:1, and t18:0) which is typically found in plasma, skin, brain, d16:1 which is found in bovine milk and d20:1 sphingoid is found in the brain as well (Pruett et al., 2008). The fatty acid attached to sphingoid backbone found in mammalian system commonly range from 14 to 36 carbon and are saturated or have a single double bond and with or without hydroxyl group. It is important to consider these differences when probing the GlcCer pathways but to also consider that similar but not identical lipid mediators can regulate the same pathways in different organisms.

**TABLE 1:** Major biosynthesis enzymes involved in the production of glucosylceramides. The pathway for synthesizing glucosylceramides is conserved in *C elegans* and mammalian system. The table summarizes the major enzymes involved in the production of glucosylceramides in *C. elegans* and their mammalian homologs (Pralhada Rao et al., 2013; Quinville et al., 2021).

Enzyme	*C. elegans* homolog	Mammalian homolog	Function
Fatty acid synthase	FASN-1	FAS	Catalyzes the *de novo* production of fatty acids from acetyl-CoA
Serine palmitolyl transferase	SPTL-1,2,3	SPTLC/SPT	Synthesizes sphingoid backbone from the addition of an amino acid (e.g., Serine) to a fatty acid (e.g., C15iso)
Ceramide Synthase	HYL-1,2 and LAGR-1	CerS	Synthesizes ceramides by adding a fatty acid to a sphingoid base
Ceramide glucosyl transferase	CGT-1,2,3	GCS/UGCG	Produces glucosylceramide by adding a UDP-glucose to an existing ceramide molecule

## Analysis of glucosylceramides by mass spectrometry

The high structural similarity of GlcCer species makes mass spectrometry an ideal analytical technique used to identify and characterize this species. The advancement of high-resolution mass spectrometry allows for the detailed structural characterization of GlcCer species based on their intact mass/charge ratio and fragmentation pattern. GlcCer can ionize both in the positive and negative ion mode but typically the ionized form used for quantitative analysis are the proton adducts [M+H] of GlcCer measured in the positive ion mode (Shaner et al., 2009; Peng et al., 2017). Using stable isotope labeling technique of various amino acids, Hannich et al. (2017), found all GlcCer species contained a d17iso sphingoid base. In addition, feeding worms with different sphingoid base apart in the absence of d17iso sphingoid base leads to severe developmental and digestive defects suggesting that d17iso is the major if not the exclusive sphingoid base incorporated. Because the sphingoid base in *C. elegans* is a C17iso sphingoid base, the fragmentation of sphingolipid species generates a unique product with m/z 250.25 (Hannich et al., 2017; Cheng et al., 2019; Hänel et al., 2019) (Figure 1B). Thus, the N-fatty acyl chain can easily be predicted using the intact m/z of a GlcCer species and fragment product of the sphingoid base.

GlcCer can be detected by directly injecting purified samples in mass spectrometry, but often mass spectrometry is combined with separation techniques such as liquid chromatography (LC) to improve the detection of GlcCer. Liquid chromatography is useful for separating GlcCer species from other sphingolipid species and for separating individual GlcCer species based on their chain length, degree of saturation, and number of hydroxylated groups present (Merrill, 2011; Peng et al., 2017). The separation of individual species improves the sensitivity of the detection of GlcCer by overcoming challenges such as ionization suppression. In general, reverse phase LC (RP-LC) is useful for separating different species of GlcCer and GlcCer from other sphingolipids. However, RP-LC cannot separate GlcCer from its isomer galactosylceramide (GalCer) and to overcome this challenge normal phase chromatography is used to separate these isomeric hexosylceramides (Shaner et al., 2009). In *C. elegans* however, there is no report of the presence of GalCer and most studies have reported hexosylceramides in the nematode as GlcCer (Marza et al., 2009; Mosbech et al., 2013; Zhu et al., 2013; Cheng et al., 2019; Wang et al., 2021). Before analyzing GlcCer by the analytical techniques outlined above, lipids are first purified from the biological samples. An effective method for extracting sphingolipids is either by the classical chloroform/methanol extraction or more recently the methyl tert-butyl ether (MTBE) extraction (Shaner et al., 2009). The challenge with these methods is that they extract all lipid classes present within the sample which can often influence the efficient chromatographic separation of GlcCer. An alkaline hydrolysis step is often carried out after CHCl_3_/MeOH or MTBE extraction to hydrolyze all ester bonds thereby, eliminating all phospholipids and glycerolipids that are present in the sample and improving the sensitivity of measuring GlcCer (Figure 1C) (Peng et al., 2017; Cheng et al., 2019; Hänel et al., 2019).

**FIGURE 2:**
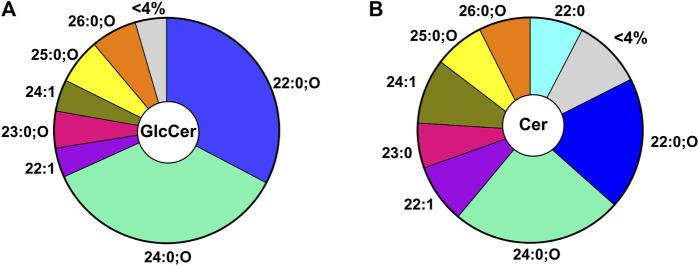
Distribution of GlcCer **(A)** Distribution of most abundant GlcCer (>4% of total GlcCer) in WT young adult worms. Here the major GlcCer species are 22:0;O and 24:0;O GlcCer which account for ∼33% and 36% of the total pool of GlcCer **(B)** Distribution of the most abundant ceramides (>4% of total ceramide) in WT young adult worms. The major ceramide species are the 22:0;O and 24:0;O Cer species which account for ∼44% of the total Cer pool.

The GlcCer pool in *C. elegans* is generated from 7 major species (i.e., comprises more than 4% of the pool). Because the detected GlcCer species all contain a d17iso sphingoid base, the diversity is generated from the N-acyl chain (Figure 2A). In nematodes, the GlcCer species contain acyl chain lengths ranging from 20–27 with 0 or 1 double bonds and with or without an additional hydroxyl group(s) (Cheng et al., 2019; Hänel et al., 2019; Xatse et al., 2023). Interestingly, two main GlcCers constitute the majority of the GlcCer pool: specifically, the 22:0;O GlcCer and 24:0;O GlcCer make up ∼33% and 36% respectively of the total GlcCer pool (Figure 2A) (Xatse et al., 2023). The particular distribution of GlcCer species indicates the specific regulation of lipids and suggests particular roles for individual species. Intriguingly, this predominance is also reflected, albeit to a lesser extent, in the direct precursors of these GlcCer in the ceramide pool, with 22:0;O Cer and 24:0; O Cer making up ∼19% and 25% of the total ceramide population (Figure 2B). Although the overall distribution of Cer is similar to the GlcCer pool, distinct differences indicate that the metabolic wiring enriches certain lipids in the GlcCer pool (e.g., 22:0;O GlcCer) while others are depleted (e.g., 22:0 GlcCer) (Xatse et al., 2023). It is not yet clear if the shift in these lipids is due to the increased synthesis of certain species or due to the consumption of other species by other intersecting metabolic pathways.

## Mass spectrometry defines a role for glccer in glucose response

Several studies have examined the phenotypic effects of impaired GlcCer synthesis and have found roles for GlcCer in apical membrane polarity, tubulogenesis, and larval development (Zhang et al., 2011; Gobel et al., 2012; Zhu et al., 2015; Zhu et al., 2021; Li et al., 2022). Recently, we established that in response to stress, *C. elegans* requires a compensatory shift in lipid metabolic pathways. Specifically, when young animals are fed glucose, their membrane composition remains fairly stable with few statistically significant changes (Vieira et al., 2022). However, using ^13^C labeling strategies that label all dietary carbons following glucose stress, there is an increased influx of new C17iso fatty acids into the membrane. To further investigate whether this influx of C17iso into the membrane was critical for surviving glucose stress, we observed that reducing the production of mmBCFA by knocking down ELO-5 leads to premature death in the nematodes on high glucose diet (Vieira et al., 2022). This C17iso is exclusively synthesized in the nematode as the bacterial diet does not contain any mmBCFAs. Because of the defined role of mmBCFAs in generating the precursors for GlcCer, we specifically quantified the Cer and GlcCer populations in animals exposed to high levels of glucose (Xatse et al., 2023).

When WT animals were fed with 100 mM glucose, ceramide, and GlcCer profiles were relatively stable except for a significant increase in the level of the 22:0;O GlcCer species. It is important to consider that 100 mM is a higher amount of glucose than used in many studies. In these studies, this elevated stress was used to maximize any perturbations in lipid metabolism but other concentrations showed similar impacts of the lipids (Vieira et al., 2022; unpublished data). Interestingly compromising ELO-5 leads to a significant decrease in overall levels of this 22:0;O GlcCer. Taken together, this study implicated a role for GlcCer and specifically for the 22:0;O GlcCer species in regulating this response (Xatse et al., 2023). To our knowledge, glucose is the only stress where the GlcCer population has been comprehensively monitored in the nematode; however, other stressors are likely to cause perturbation and should be examined using similar methods. The same 22:0; O GlcCer has been connected to TOR (Target of Rapamycin) signaling (Zhu et al., 2013). The TOR complex is a serine/threonine kinase in *C. elegans* and conserved in mammalian organisms and is made up of two main complexes, TORC1 and TORC2 (Saxton and Sabatini, 2017). An unbiased genetic screening in *C. elegans* identified GlcCer as an important regulator of TORC1 activation (Zhu et al., 2013). This GlcCer/TORC1 signaling pathway is proposed to be a metabolic checkpoint in the intestine of the nematodes that coordinates post-embryonic growth and development (Zhu et al., 2013).

A recent study identified a role for GlcCer in TOR regulation in a *C. elegans* aging model (Wang et al., 2021). In this study, long-lived germline deficient worms (*glp-1*) were shown to exhibit significantly upregulated levels of the sphingolipid synthesis enzyme ELO-3. ELO-3 is an enzyme involved in the synthesis of saturated fatty acids with chains longer than twenty carbons that are incorporated into the sphingoid base in *C. elegans* to produce ceramides and GlcCer. Further investigation revealed a specific GlcCer species (C22 GlcCer) as the sphingolipid critical for *glp-1* mediated lifespan extension. C22 GlcCer modulates lifespan extension by regulating clathrin membrane localization and lysosome homeostasis resulting in the activation of the transcription factor SKN-1. Activation of SKN-1 leads to the suppression of TOR and the subsequent increase in the lifespan of *glp-1* animals. The contrasting role of GlcCer in TOR activation is intriguing as it suggests that the regulation may depend on the stage of development of the organism and perhaps the type of acyl chain attached to GlcCer. The idea of acyl chain specificity in regulating TOR activation is not surprising as previous studies show that the acyl chain length of sphingolipids expressed in distinct tissues or cellular compartments can perform unique functions. Thus, it will be interesting to further investigate the detailed role of acyl chain length specificity of GlcCer in TOR regulation.

Furthermore, we found that compromising the GlcCer production by either *cgt-3* and *cgt-1/cgt-3* RNAi results in a dramatic reduction in lifespan of animals on glucose demonstrating a novel role for GlcCer in surviving glucose stress (Xatse et al., 2023). To further corroborate that the shortened lifespan of animals with ELO-5 knockdown was due to compromised GlcCer production, we supplemented *elo-5* RNAi with sphingolipid extract from WT animals. Interestingly, supplementing *elo-5* RNAi-treated animals with WT sphingolipid extract rescues the shortened lifespan under glucose stress. In addition, when the GlcCer profile of *elo-5* RNAi supplemented with WT sphingolipid extract is measured, there are significant shifts in GlcCer levels towards WT GlcCer levels including a significant surge in the levels of the 22:0;O GlcCer species (Xatse et al., 2023).

## Challenges and perspectives

One of the major challenges for the interrogation of lipid metabolism is the confirmation of genetic studies. A potential avenue to validate these conclusions for metabolomic studies is to supplement the product of the pathways to show rescue. There are some challenges to the supplementation of lipids through the diet. First, lipids must be digested in the intestine of the animals which involves the hydrolysis of the lipid into their constituents (Watts and Ristow, 2017). Therefore, the supplementation may not be of the intact lipid you are looking to probe. It is important to measure the lipid composition of the animals following supplementation. In the sphingolipid supplementation protocols, we quantified an increase in specific GlcCer species, but it is not possible to distinguish whether this was due to the provision of that lipid or increased production of that lipid as a result of increased availability of individual components.

It is also important to consider the interconnectedness of metabolic pathways. Many lipids have multiple fates in the animal and changes in lipid abundance may reflect increased synthesis of a lipid or decreased consumption. To distinguish these possibilities, our group is developing stable isotope tracing methods to monitor dynamics in addition to the overall abundance of each species (Perez and Van Gilst, 2008; Drechsler et al., 2016; Sultana and Olsen, 2020).

## Conclusion

The loss of GlcCer synthesis enzymes with mutation or RNAi demonstrates a critical role for GlcCer metabolism in development and in the response to elevated glucose diets. Mass spectrometry allows for the quantification of all the individual GlcCer species along with the precursor and product populations. This combination allows for the detection of each species to define which specific pathways are responsible for biological activities. Ultimately, these studies will increase the knowledge of how sphingolipid metabolism can be modulated to ameliorate disease states.
